# Immunogenicity of Type IV Pilin Proteins from *Clostridium perfringens* in Chickens

**DOI:** 10.3390/microorganisms13010120

**Published:** 2025-01-09

**Authors:** Audrey Charlebois, Nicolas Deslauriers, Lila Maduro, Martine Boulianne

**Affiliations:** Chaire en recherche avicole et Centre de Recherche en Infectiologie Porcine et Aviaire (CRIPA), Département de sciences cliniques, Faculté de Médecine Vétérinaire, Université de Montréal, Saint-Hyacinthe, QC J2S 2M2, Canada; audrey.charlebois@umontreal.ca (A.C.); nicolas.deslauriers.1@umontreal.ca (N.D.); lila.maduro@umontreal.ca (L.M.)

**Keywords:** pilus, poultry, necrotic enteritis, vaccine

## Abstract

*Clostridium perfringens*, the causative agent of necrotic enteritis in chickens, is controlled by in-feed antibiotics. With increasing pressure to reduce antimicrobial use, the development of alternative preventive tools is needed. Type IV pili proteins have been shown to be immunogenic in many Gram-positive bacteria. The aims of this study were to evaluate the immunogenic potential of pilins (PilA1, PilA2 and PilA3) from *C. perfringens* in chickens and to verify their ability to protect against necrotic enteritis. Chickens were immunized twice with 50 µg of recombinant proteins and adjuvant, resulting in a good and specific serum antibody response. Next, one-day-old chicks were injected three times with the same vaccines, and then infected with *C. perfringens*. Mean OD_450_ values ten times higher than the controls were obtained for IgY (*p* < 0.05) and a significantly lower cecal count of *C. perfringens* was observed in the birds injected with PilA3. However, no reduction in the severity of intestinal lesions was observed. All three pilin proteins were shown to be highly immunogenic in the chickens. Although immunization with the pilins did not protect the birds against necrotic enteritis in this study, it was interesting to observe that vaccination with the recombinant PilA3 protein reduced *C. perfringens* cecal colonization.

## 1. Introduction

Antibiotics are used as feed additives to prevent and control necrotic enteritis, a fatal disease in poultry caused by *Clostridium perfringens* [1]. However, this practice has come under increasing scrutiny due to the potential development of antibiotic-resistant human pathogenic bacteria [2]. Following the ban on antibiotic feed additives in Europe, the incidence of necrotic enteritis has increased in these countries [3]. However, increasing consumer pressure is pushing animal production to eliminate, or at least significantly reduce, the use of antibiotics. The control of necrotic enteritis is critical for sustainable antibiotic-free chicken production, but no effective and fully preventive vaccine has been discovered [1].

*Clostridium perfringens* is a Gram-positive, anaerobic spore-forming bacterium that causes a wide variety of diseases in humans and animals. This microorganism is a commensal of the gastrointestinal tract of mammals and is also commonly found in soil and water [4]. Similarly, *C. perfringens* is known to be a normal intestinal resident in chickens, with commensal and pathogenic strains likely coexisting. The proliferation of pathogenic strains of *C. perfringens* and the resulting necrotic enteritis occur when this homeostasis is disrupted by one or more predisposing factors. For example, coccidiosis, environmental stressors, high dietary levels of non-starch polysaccharides will cause damage to the epithelium, increase intraluminal nutrient availability and excessive mucus production, impair peristalsis and feed passage, and thus provide an optimal environment for *C. perfringens* to multiply [1]. Toxins were long thought to be the virulence factors responsible for the pathogenicity of *C. perfringens*, with the alpha toxin being the first culprit, until the discovery of the NetB toxin and its role in the *C. perfringens* virulence [5]. This discovery led to a new classification scheme based on the production of six toxins (alpha, beta, epsilon, iota, enterotoxin and NetB) and divided the *C. perfringens* into seven toxigenic biotypes (A to G) [6]. Pathogenic strains of *C. perfringens* responsible for necrotic enteritis are now considered to be type G *C. perfringens*. However, recent work using a ligated loop model has shown that *netB*-negative strains are also associated with necrotic enteritis lesions [7]. The histologic examination of early necrotic enteritis lesions has shown that *C. perfringens* rods adhere to the intestinal villi, causing a heterophilic inflammatory response and early damage to the villus tips [8]. This observation has led to the hypothesis that the ability of *C. perfringens* to adhere to enterocytes is an important initiating factor in the cascade of pathological changes leading to necrotic enteritis. This hypothesis is supported by the work of Wade et al. [9] and Lepp et al. [10], who identified a putative collagen adhesion gene, *cnaA*, and predicted pilin subunits, *FimA* and *FimB*, respectively, in disease-causing *C. perfringens* strains. The importance of adherence in early bacterial pathogenesis is well known and is usually mediated by different bacterial surface structures such as adhesin, fimbria and pilis.

Type IV pili are bacterial surface-exposed fibers that mediate adherence, colonization, DNA transfer, protein secretion and twitching motility, among other functions [11,12]. The type IV pili structural subunits are called pilins but other proteins with structures similar to pilins, called pilin-like proteins or minor pilins, are involved in pilus biogenesis and dynamics [11]. Pili subunits, such as PilA1, PilA2 and PilA3, from the Gram-positive bacteria *Clostridium difficile*, have been shown to be immunogenic [13]. The facts that pili are present on the surface of bacteria, consist of many identical repeating subunits and are expressed in the early stages of infection make them interesting as potential vaccine targets. Previous studies have shown that immunization with type IV pili subunits or whole pili can confer protection against *Vibrio cholera*, *Moraxella bovis* and *Dichelobacter nodosus* [14,15,16,17]. A veterinary vaccine consisting of whole *M. bovis* pili is now commercially available in the USA (Piliguard^®^ Pinkeye TriView, Merck Animal Health). A vaccine directed against the *C. perfringens* type IV pilins may prove effective in preventing the colonization of this microorganism in the intestines of poultry and subsequent development of necrotic enteritis.

Genes encoding type IV pili have been identified in *C. perfringens* genomes and pilins have been detected at the surface of bacterial cells by immunogold labeling and indirect immunofluorescence [18]. However, their immunogenicity is unknown. The aims of this study were to evaluate the immunogenic potential of three pilins from *C. perfringens* in chickens and to verify their ability to protect against necrotic enteritis.

## 2. Materials and Methods

### 2.1. Plasmid Construction, Escherichia coli M15 Transformation and Protein Expression

DNA sequences of three pilin genes from *C. perfringens* CP0994 (*pilA1* and *pilA3*) and CP1876 (*pilA2*) lacking the codons for the signal peptide and the N-terminal hydrophobic domain were amplified by PCR with primers containing restriction sites for BamHI, HindIII or PstI (Table 1). Two strains of *C. perfringens*, both belonging to the microbial collection of the Chair in Poultry Research and isolated from chickens presenting lesions of necrotic enteritis, were used because we were unable to amplify the *pilA2* gene from the strain CP0994. The PCR products were digested with the enzyme BamHI and HindIII or PstI (New England Biolabs, Whitby, ON, Canada) and ligated into similarly digested pQE-30 expression vector (Qiagen, Toronto, ON, Canada). This placed the pilin genes immediately downstream of the hexa-histidine tag sequence. Competent *E. coli* M15 cells (Qiagen) were transformed with each of the recombinant pQE-30 vectors and plated on LB agar (Thermo Fisher Scientific, Nepean, ON, Canada) containing 25 µg/mL kanamycin and 100 µg/mL ampicillin (Sigma-Aldrich, Oakville, ON, Canada). The integrities of the cloned sequences were checked by DNA sequencing. Protein expression was carried out following the manufacturer’s instructions [19].

### 2.2. Protein Purification

Cell pellets were thawed and resuspended in wash buffer (50 mM NaH_2_PO_4_, 300 mM NaCl, 20 mM imidazole, pH 8.0) supplemented with 1 mg/mL of lysozyme (Sigma-Aldrich). The pellets were incubated on ice for 30 min and then sonicated. The lysates were centrifuged at 35,000× *g* for 30 min at 4 °C. Nickel-nitrilotriacetic acid (Ni/NTA) affinity purification was performed on an AKTA Xpress FPLC system using 1 mL HisTrap HP columns (GE Healthcare, London, ON, Canada). After washing, the protein was eluted from the columns with increasing concentrations of imidazole (1–50%) in elution buffer (50 mM NaH_2_PO_4_, 300 mM NaCl, pH 8.0). Samples were collected at various time points and loaded onto SDS-PAGE gels to assess the purity of the target recombinant proteins. Protein concentrations were measured using the BCA Protein Assay Kit (Thermo Fisher Scientific).

### 2.3. Immunogenicity of Pilin Proteins in Chicken

Twenty-two 8-week-old specific-pathogen-free hens were purchased from the Canadian Food Inspection Agency (Nepean, ON, Canada) and housed at the Poultry Research Centre. Fecal samples were cultured for *C. perfringens* before the start of the experiments to confirm that the chickens were free of *C. perfringens*. The birds were individually identified with a wing tag and randomly allotted to their respective group. All the birds were provided with a commercial broiler diet and water ad libitum and given a one-week acclimatization period prior to immunization. Blood samples were collected as a pre-immunization control before injection. For immunization, the hens were injected intramuscularly with a mix of 50 µg of pilin proteins resuspended in sterile phosphate-buffered saline (PBS) [20,21] and Freund’s incomplete adjuvant (FIA; Thermo Fisher Scientific). The hens from the control group were immunized with PBS and FIA only. After 3 weeks, the hens were immunized again following the same protocol. At 15 weeks, the hens were sedated with an intramuscular injection in the pectoral muscle with a combination of ketamine (25 mg/kg) and xylazine (2.5 mg/kg) and were then euthanized by cervical dislocation. Blood and ileo-cecal samples were collected for ELISA analysis of IgY and IgA, respectively. All the chickens were handled according to the ethical guidelines of the Canadian Council on Animal Care for the duration of the experimental trial. They were examined daily during the trial and twice daily for the five days following immunization, with special attention to the injection site. The protocol was approved by the Ethics and Animal Use Committee of the Université de Montréal (17-RECH-1895).

### 2.4. Measurement of Antibody Titers (IgY and IgA)

Specific antibody titers were determined by the end-point dilution method using an enzyme-linked immunosorbent assay (ELISA). Microtiter plates (Nunc, Thermo Fisher Scientific) were coated with the three pilin proteins (5 µg/mL in 0.1 M carbonate buffer, pH 9.6) for 60 min at 37 °C, followed by an overnight incubation at 4 °C. After the coated plates were blocked for 60 min at room temperature with PBS containing 3% bovine serum albumin (BSA; Sigma-Aldrich), sera from the immunized birds, together with sera from the control birds (or ileo-cecal mucosal scrapings for IgA), were serially diluted in PBS containing 1% BSA and incubated for 2 h with recombinant protein-coated plates at room temperature. After the plates were washed with PBS containing 0.1% Tween 20 (PBST), peroxidase-conjugated goat anti-chicken IgY (Thermo Fisher Scientific, #A16054; diluted 1:40,000 in PBST containing 1% BSA) or peroxidase-conjugated goat anti-chicken IgA (Thermo Fisher Scientific, #PA1-84679; diluted 1:5000 in PBST containing 1% BSA) was added to the microplates and the mixture was incubated for 60 min at room temperature. After washing the plates with PBST, the color reaction was developed by using a TMB solution (Thermo Fisher Scientific, #002023). The reaction was stopped by the addition of 0.18 M H_2_SO_4_. Absorbance at 450 nm was measured in an ELISA spectrophotometer (Bio Tek Instruments, Winooski, VT, USA).

### 2.5. SDS-PAGE and Immunoblotting Procedure

For the SDS-PAGE, protein samples were diluted in 5X sample buffer (1 M Tris, pH 6.8, 10% sodium dodecyl sulfate (SDS), 50% glycerol, 100 mM DTT, 0.01% bromophenol blue). An appropriate quantity of each protein and molecular weight marker (Low-Range Rainbow Molecular Weight Marker, GE Healthcare) was then loaded into 12.5% bis-acrylamide gels. The gels were run at room temperature in running buffer (25 mM Tris, 0.2 M glycine, 0.1% SDS) at 100 V/10 min and then 200 V until the dye front reached the bottom of the gel cassette. After the SDS-PAGE, the proteins were transferred to nitrocellulose membranes (Bio-Rad, Mississauga, ON, Canada) using transfer buffer (25 mM Tris-base, 192 mM glycine, 20% methanol) by running at 100 V for 1 h. The membranes were blocked in Tris-buffered saline (TBS) containing 2% (wt/vol) skim milk powder for 1 h at room temperature and then incubated overnight at 4 °C with serums (1:1000 dilution) in the blocking buffer (TBS containing 2% skim milk). After three washes in TBS, the membranes were incubated for 1 h at room temperature with peroxidase-conjugated goat anti-chicken IgY (GE Healthcare). After being washed three times, the membranes were developed by incubation in a TMB solution (Thermo Fisher Scientific) in the dark.

### 2.6. Experimental NE Disease Model

One hundred and three 1-day old mixed specific-pathogen-free chicks were purchased from the Canadian Food Inspection Agency (Nepean, Canada) and housed at the Poultry Research Centre. Fecal samples were cultured for *C. perfringens* prior to starting the trial to confirm that the chicks were free of *C. perfringens*. The birds were weighed, individually identified with a wing tag and randomly allotted to their respective group. The chicks were provided with an anticoccidial and antibiotic-free commercial broiler ration containing 25% wheat with no β-glucanase enzymes added, as a risk factor for NE, and water ad libitum. Before the first vaccination (day 6), blood samples were obtained as a pre-immunization control, and the chicks were weighed. For immunization, as previously described in other studies [20,21], the birds were injected subcutaneously with 50 µg of pilin proteins in sterile phosphate-buffered saline (PBS) and Freund’s incomplete adjuvant (FIA; Thermo Fisher Scientific). The birds from the control group were immunized with PBS and FIA only. At days 12 and 17, the chickens were immunized and weighed again following the same protocol. Blood samples were also obtained at day 17. The experimental NE disease model was as previously described [22]. Briefly, at day 14, all the groups received 10 times the dose of a coccidiosis vaccine (Coccivac^®^-B52, Merck Animal Health, Kirkland, QC, Canada) as another risk factor for NE [23,24]. At day 21, half the birds were weighed and sacrificed. The chickens were sedated with an intramuscular injection in the pectoral muscle with a combination of ketamine (25 mg/kg) and xylazine (2.5 mg/kg) and were then euthanized by cervical dislocation. Blood and ileo-cecal samples were obtained for ELISA analysis of IgY and IgA, respectively. Cecal contents were also retrieved for *C. perfringens* detection and quantification. On day 21, 22 and 23, the remaining birds were orally administered, 4 times each 12 h, with 10^7^ cfu/mL of a mix of 3 pathogenic *C. perfringens* strains (CP1876, CP1884 and CP1885). All the birds were euthanized on day 24 and samples were collected as described before. Macroscopic examinations and NE lesion scoring were achieved on the euthanized birds based on the Prescott grid [25] while *Eimeria* lesions, if present, were scored according to the Johnson and Reid scale [26], all by a poultry pathologist blinded to group allocation. All the chickens were handled according to the ethics guidelines of the Canadian Council on Animal Care for the duration of the experimental trial. To safeguard animal welfare, scientific endpoints, humane intervention points and monitoring procedure were part of a protocol approved by the Ethics and Animal Use Committee of the Université de Montréal (19-RECH-2006).

### 2.7. Quantification of C. perfringens in Ceca

DNA was extracted from the cecal contents with the Isolate fecal DNA kit (FroggaBio Inc., Toronto, ON, Canada). A real-time PCR was used to quantify *C. perfringens* in those samples. The primers and probe used were previously described by Albini et al. [27]. Each PCR reaction of 20 mL contained 10 µL of TaqMan Fast Advanced Master Mix (Thermo Fisher Scientific), 300 nM of each forward and reverse primer, 150 nM of probe (Thermo Fisher Scientific) and 2 µL of DNA sample (diluted 1:10 in nuclease-free water). The PCRs were carried out in a ViiA 7 Real-time PCR System (Applied Biosystems, Thermo Fischer Scientific) with the standard thermal cycle protocol used. The quantitation of *C. perfringens* was carried out by comparison to a standard curve.

### 2.8. DNA Extraction and Whole Genome Sequencing

Genomic DNA was extracted from the overnight cultures using the phenol chloroform extraction method [28]. The DNA quality and quantity were assessed using a Nanodrop^TM^ spectrophotometer (ThermoFisher Scientific) using A260/280 and A260/A230 ratios. The DNA extractions were sent to the Genome Quebec sequencing center (Génome Québec Inc., Montréal, QC, Canada) and whole genome sequencing was performed using an Illumina MiSeq instrument (Illumina, San Diego, CA, USA).

### 2.9. Bioinformatic Analysis

The genomic DNA reads were trimmed to remove adapters and poor-quality data using the bioinformatics tools Scythe v.0.991 [29] and Sickle v.1.33 [30]. The quality of reads was assessed before and after the trimming using FastQC v.0.11.9 [31] to ensure adequate removal. The genome assembler tool SPAdes v.3.15.2 [32] was used to de novo assemble the reads into contigs. Protein annotation was performed using Prokka v.1.14.6 [33] and BLASTp v.2.16.0 [34] was used to identify PilA proteins from genomes using the following reference sequences: PilA1 (CPE2288, GenBank accession number BAB81994.1), PilA2 (CPE2284, GenBank accession number BAB81990.1) and PilA3 (CPE2278, GenBank accession number BAB81984.1). The alignment of the PilA2 proteins from CP0994 and CP1876 to the reference PilA2 protein was performed using MUSCLE v.3.8 [35] with default parameters and was visualized using Jalview v.2.11.2.5 [36]. The homology between the protein sequences of the PilA proteins in this study and other adhesion factors such as FimA (Genbank accession#: WP_057230739.1), FimB (Genbank accession#: WP_057230742.1), CnaA (Genbank accession#: WP_057230734.1) and FBA (Genbank accession#: WP_003449129.1) evaluated using BLASTp.

### 2.10. Statistical Analysis

For the determination of the immunogenicity of the pilin proteins, the statistical differences between the pre-immune and post-immune titers for each antigen among the different vaccination groups were determined by a mixed linear model with a Benjamini–Hochberg sequential procedure. For the comparison of lesion scores between groups, the Mantel–Haenszel test was used. Following the experimental NE disease model, the statistical differences between the pre-immune and final sera titers for each antigen among the different vaccination groups were determined by a *t*-test for the dependent samples. An unequal variance *t*-test was used to determine the statistical differences between the numbers of *C. perfringens* bacterial cells before and after experimental infection.

## 3. Results

### 3.1. Protein Expression and Purification

**Figure 1:**
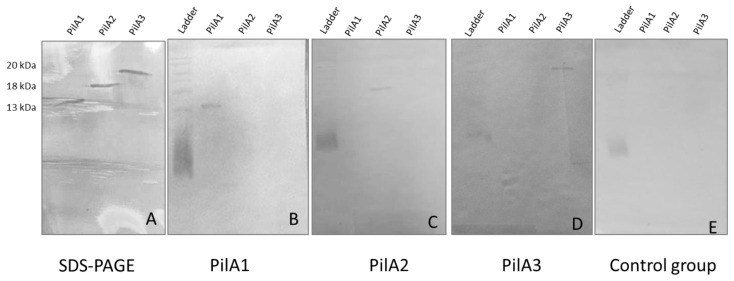
SDS-PAGE of recombinant pilus proteins and Western blot analyses: (**A**) Purified PilA1 (lane 1), PilA2 (lane 2) and PilA3 (lane 3) were visualized by SDS-PAGE and Coomassie staining. The molecular weights are indicated on the left. (**B**–**E**) The specificity of antibodies raised in chickens was determined by Western blot analyses. Sera from the group immunized with PilA1 (**B**), PilA2 (**C**), PilA3 (**D**) and from the control group (**E**) were tested against PilA1, PilA2 and PilA3.

The coding sequences of the three pilin genes from *C. perfringens* (*pilA1*, *pilA2* and *pilA3*), lacking the codons for the signal peptide and the N-terminal hydrophobic domain, were successfully amplified and cloned in *E. coli* M15. After production and purification, good amounts of expressed proteins were obtained for each pilin and the molecular weights were consistent with the predicted values (Figure 1A).

### 3.2. Immunization with Pilin Proteins and Serum Antibody Response

**Figure 2:**
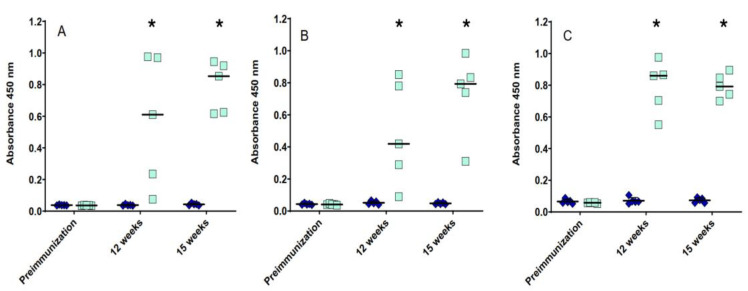
Serum IgY response against pilin proteins. Serum IgY response against PilA1 (**A**), PilA2 (**B**) and PilA3 (**C**) from birds immunized with 50 µg of recombinant proteins. Each symbol represents a single bird and the horizontal lines represent means. Statistical differences between pre-immune and post-immune absorbance values among the control group (blue diamonds) and the different vaccination groups (teal squares) were determined by a mixed linear model with a Benjamini–Hochberg sequential procedure. * *p* < 0.0001.

To test the immunogenicity of the three purified pilins, four groups of birds were immunized twice with PilA1, PilA2 or PilA3 recombinant proteins. The fourth group was immunized with adjuvant alone. Serum IgY titers against pilin proteins were determined in all five birds from each group before and after both immunizations (12 weeks and 15 weeks). Significantly higher serum antibody responses were observed at 12 (mean OD_450_ values between 0.485 and 0.791) and 15 weeks (mean OD_450_ values between 0.731 and 0.795) in all of the immunized groups compared to the pre-immune controls, with mean OD_450_ values between 0.043 and 0.073 (Figure 2). To verify that the antibody response was specific to the pilin proteins, a Western blot analysis was performed. The antibodies raised against PilA1, PilA2 and PilA3 were specific to their immunizing antigen (Figure 1B–E). As for the mucosal antibody response, no significant increase in the IgA titers was observed for each pilin protein tested.

### 3.3. Protection Against C. perfringens in an Experimental Necrotic Enteritis Model

**Figure 3:**
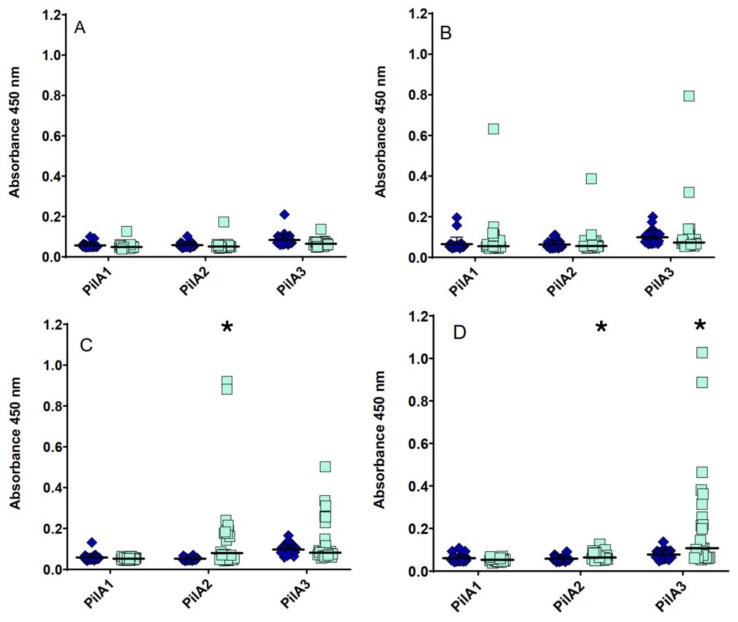
Comparison between pre-immunization and final sera IgY response against pilin proteins following the experimental *C. perfringens* infection. Comparison between pre-immunization and final sera IgY response against PilA1, PilA2 and PilA3 from the control (**A**), PilA1-immunized (**B**), PilA2-immunized (**C**) and PilA3-immunized (**D**) groups. Each symbol represents a single bird and horizontal lines represent means. Statistical differences between pre-immune (blue diamonds) and final (teal squares) absorbance values were determined by a *t*-test for dependent samples. * *p* < 0.05.

**Figure 4:**
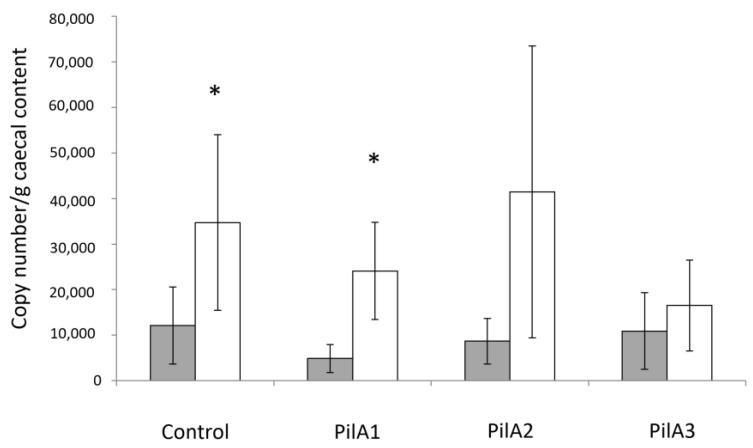
Quantification of *C. perfringens* in ceca before and after infection. Quantification of *C. perfringens* in ceca before and after experimental infection for each group as determined by real-time PCR. Statistical differences between the bacterial counts before (gray bars) and after (white bars) infection were determined by an unequal variance *t*-test. * *p* < 0.05.

The ability of the antibodies raised following immunization with PilA1, PilA2 or PilA3 to protect the birds against necrotic enteritis was assessed with an experimental necrotic enteritis model developed at the Chair in Poultry Research. No significant difference in the average NE lesion scores was observed between the immunized (PilA1: 0.45; PilA2: 0.92; PilA3: 0.67) and adjuvant-only control (Control: 0.83) groups, with all the groups displaying a low severity of lesions. Moreover, no significant difference in the average *Eimeria acervulina* (PilA1: 1.36; PilA2: 1.85; PilA3: 1.33) and *Eimeria maxima* (PilA1: 0.18; PilA2: 0.38; PilA3: 0.42) lesion scores was observed between the immunized and adjuvant-only control (Control Ea: 1.42 and Control Em: 0.17) groups. No significant difference was observed in the weight gain between the groups. A significantly higher serum antibody response was observed in the final sera in the PilA2 and PilA3 immunized groups compared to the pre-immune controls (Figure 3). As for the mucosal antibody response, no significant increase in the IgA titers was observed. Furthermore, a lower number of *C. perfringens* was observed in the ceca in the PilA3 immunized group after infection compared to the other immunized groups, with no significant difference from the non-infected group (Figure 4).

### 3.4. Comparison and Analysis of C. perfringens Pilin Sequences

**Figure 5:**
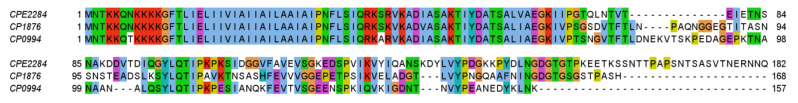
Alignment of the PilA2 protein sequences from strains CP0994 and CP1876 to the reference PilA2 protein (CPE2284). Sequences were aligned using MUSCLE v3.8 and visualized using Jalview v.2.11.2.5. Names of strains and the reference are shown on the left side of the sequences whereas 297 amino acids positions are shown above. Conserved regions are highlighted in colors. Gaps in a 298 sequence are indicated by the symbol dash (-).

To investigate the reason why the *pilA2* gene could only be amplified using strain CP1876, the alignment of PilA2 sequences of CP0994 and CP1876 were aligned to the reference PilA2 sequence (CPE2284). A total of 21 regions of different sizes were fully conserved among the three PilA2 proteins compared, whereas 17 regions were not conserved (Figure 5). To assess if the type IV pilus proteins PilA1, PilA2 and PilA3 were similar to other proteins involved in the adhesion of *C. perfringens* and whose immunogenicity in broiler chickens was evaluated (CnaA, FimA, FimB, FBA), BLASTp analyses were performed. No homology between the three PilA proteins and the other adhesion factors was observed.

## 4. Discussion

In this study, we sought to evaluate the potential immunogenicity of three pilin subunits of *C. perfringens* in broiler chickens. Previous studies have reported the crucial role of adhesion factors in the pathogenesis of *C. perfringens* as well as their immunogenic properties in chickens [9,37,38]. The protein sequences of PilA1, PilA2 and PilA3 were compared to those of other known adhesion factors of *C. perfringens* whose potential immunogenicity were assessed in vivo in broiler chickens. The results indicated that the PilA proteins were different and highlighted the diversity of adhesion factors found in *C. perfringens* strains. Interestingly, the *pilA2* gene could not be amplified using strain CP0994 prior to the cloning in the expression vector as were the *pilA1* and *pilA3* genes. However, the amplification of *pilA2* could be achieved using strain CP1876, which suggested a possible deletion of the *pilA2* gene in strain CP0994. The bioinformatics analysis revealed that both strains had the PilA2 protein but that differences in their nucleotides and amino acid sequences could be observed. As the primers were developed based on the reference PilA2 sequence and because the sequence of PilA2 did not appear to be conserved among the *C. perfringens* strains in this study, it was hypothesized that the designed primers failed to anneal properly to the DNA target, hence the need to use another strain [39]. PilA2 is one of the two major pilins forming the type IV pili in *C. perfringens* [18] and, as such, is continuously exposed to the environment and immune system. The observed genetic variation in this protein could be part of an antigenic variation mechanism to avoid detection by the immune system, as seen in other bacteria [40].

In the present study, for the *C. perfringens* PilA1, PilA2 and PilA3 pilin proteins, the mean OD_450_ values obtained for IgY were significantly higher for the three pilin proteins compared to the control groups. Moreover, the serum antibody responses were specific as they were only interacting with their specific protein. This immunogenicity was also observed in other studies for other pilin proteins [13,21,37,41]. The following part of this study was to evaluate the ability of these antibodies to protect birds from *C. perfringens* and necrotic enteritis. No significant difference in the average lesion scores was observed between the immunized and adjuvant-only control groups. The general low severity of the lesions detected could be due to a problem with the ambient temperature experienced at the chicks’ arrival. Indeed, for the first few days, the birds were exposed to temperatures around 35 °C because of a mechanical problem in the animal housing facilities. Many negative impacts have been associated with heat stress in poultry, including reduced growth and feed efficiency. In this study, the birds affected by heat stress in the first days of life had lower body weights than expected, hence possibly decreasing their exposure to an important predisposing factor i.e., a diet with wheat, which could have led to a low *C. perfringens* colonization, hence having an overall effect on the clinical manifestation of necrotic enteritis. Immune function can also be affected by heat stress, where one important clinical manifestation is reduced humoral immunity, which can limit the efficacy of vaccination [42,43]. Indeed, when comparing the serum antibody response between the two in vivo experiments, the absorbance values were lower in the last experiment, even though a significantly higher serum antibody response was observed in the final sera in the PilA2 and PilA3 immunized groups compared to the pre-immune controls. Another point that could have affected the necrotic enteritis scores was the low severity of the lesions due to *Eimeria*. Indeed, damages caused by *Eimeria* infection are an important predisposing factor for necrotic enteritis, allowing for *C. perfringens* growth and toxin production via plasma protein leakage and mucogenesis [44].

Even though no significant difference in the average lesion scores was observed between the immunized and adjuvant-only control groups, a lower number of *C. perfringens* was observed in the ceca of the PilA3 immunized birds after infection, indicating a lower colonization capacity. In *C. perfringens*, the PilA3 protein is considered a minor pilin [11]. Minor pilins can be implicated in the cell wall anchoring of the type IV pilus, can be part of the pilus structure and can be involved in the intracellular adhesion and interaction with host cells [45,46]. However, antibodies directed towards those proteins would hinder many functions such as motility and adhesion of the bacterial cells, which might explain the observed lower cecal *C. perfringens* counts. Because the pathogenesis of necrotic enteritis is not fully understood, other virulence factors may have contributed to the development of the lesions in the immunized birds. In the present study, following the experimental infection, it was observed that the serum antibody response and *C. perfringens* counts in the PilA2 vaccinated group were highly variable. This could be explained by the fact that the amino acid sequences of the PilA2 proteins in *C. perfringens* are much more variable than the PilA1 and PilA3 pilins [11]. It was also noted, in the second experimentation, that few birds in the PilA1 and PilA2 vaccinated groups had unspecific antibody responses (Figure 4). This might be due to the presence of the His-tag in the recombinant proteins used to vaccinate the birds. To eliminate these unspecific responses, the removal of the His-tag would be important in future studies.

Even though the vaccination with PilA1, PilA2 or PilA3 did not protect the birds against necrotic enteritis in this study, it was interesting to observe that the vaccination with the PilA3 recombinant protein, however, appears to decrease *C. perfringens* cecal colonization. Future use of a mix of antigens in the vaccine could also be an interesting avenue to investigate. Indeed, many *C. perfringens* proteins and toxins, such as the NetB toxin, iron protein or other pilus subunits, have been evaluated as potential vaccines against necrotic enteritis in chickens [21,37]. Interestingly, partial protection against necrotic enteritis was also observed in these studies. A combination of these antigens aiming at different targets of *C. perfringens* would be interesting to test in an experimental necrotic enteritis model. Genomics and immunoinformatics [47] might provide the necessary tools to construct such multi-epitope vaccines in the future.

## 5. Conclusions

In conclusion, this study showed that OD_450_ values were ten times higher than the controls for birds vaccinated with PilA1, PilA2 and PilA3 pilin proteins and that the antibodies raised against these proteins were specific. Even though the vaccination with the pilins did not protect the birds against necrotic enteritis in this study, it was interesting to observe that vaccination with the PilA3 recombinant protein appears to decrease *C. perfringens* cecal colonization. The reduction of colonization by *C. perfringens* could reduce the probability of necrotic enteritis lesion development in chickens as this bacterium needs to attach to intestinal villi and produce toxins to induce the disease. However, additional work needs to be conducted on the potential of a multi-epitope composite vaccine, including PilA3 and other target proteins, to fully protect chickens against necrotic enteritis.

## Figures and Tables

**Table 1:** Primers used in this study.

Target	Primers Sequences (5′-3′)
*pilA1* (BamHi-HinDIII)	F: TGGCTGGGATCCTATGTTAAGGATAGCGCTAA R: TGGATGAAGCTTTTATACTATAGACACCGTAA
*pilA2* (BamHi-HinDIII)	F: TGGATGGGATCCTCAATTCAAAGAAAATCAAG R: TGGCTGAAGCTTCTATTGATTATTTCTTTCAT
*pilA3* (BamHi-PstI)	F: TGGATGGGATCCTCAAATTATGTAACTTTAGA R: TGGATGCTGCAGTTATTTTTTATTTCTAAAAG

## Data Availability

The data that support the findings of this study are available from the corresponding author, MB, upon reasonable request.
